# The angiostatic peptide endostatin enhances mortality risk prediction in pulmonary arterial hypertension

**DOI:** 10.1183/23120541.00378-2021

**Published:** 2021-10-11

**Authors:** Catherine E. Simpson, Megan Griffiths, Jun Yang, Melanie K. Nies, R. Dhananjay Vaidya, Stephanie Brandal, Lisa J. Martin, Michael W. Pauciulo, Katie A. Lutz, Anna W. Coleman, Eric D. Austin, D. Dunbar Ivy, William C. Nichols, Allen D. Everett, Paul M. Hassoun, Rachel L. Damico

**Affiliations:** 1 Dept of Medicine, Division of Pulmonary and Critical Care Medicine, Johns Hopkins University, Baltimore, MD, USA; 2 Dept of Pediatrics, Division of Pediatric Cardiology, Johns Hopkins University, Baltimore, MD, USA; 3 Dept of Medicine, Division of General Internal Medicine, Johns Hopkins University, Baltimore, MD, USA; 4 Dept of Pediatrics, Division of Human Genetics, Cincinnati Children's Hospital Medical Center and University of Cincinnati College of Medicine, Cincinnati, OH, USA; 5 Dept of Pediatrics, Division of Allergy, Immunology, and Pulmonary Medicine, Vanderbilt University, Nashville, TN, USA; 6 Dept of Pediatric Cardiology, Children's Hospital Colorado, Denver, CO, USA

## Abstract

Currently available noninvasive markers for assessing disease severity and mortality risk in pulmonary arterial hypertension (PAH) are unrelated to fundamental disease biology. Endostatin, an angiostatic peptide known to inhibit pulmonary artery endothelial cell migration, proliferation and survival *in vitro*, has been linked to adverse haemodynamics and shortened survival in small PAH cohorts. This observational cohort study sought to assess: 1) the prognostic performance of circulating endostatin levels in a large, multicentre PAH cohort; and 2) the added value gained by incorporating endostatin into existing PAH risk prediction models.

Endostatin ELISAs were performed on enrolment samples collected from 2017 PAH subjects with detailed clinical data, including survival times. Endostatin associations with clinical variables, including survival, were examined using multivariable regression and Cox proportional hazards models. Extended survival models including endostatin were compared to null models based on the REVEAL risk prediction tool and European Society of Cardiology/European Respiratory Society (ESC/ERS) low-risk criteria using likelihood ratio tests, Akaike and Bayesian information criteria and C-statistics.

Higher endostatin was associated with higher right atrial pressure, mean pulmonary arterial pressure and pulmonary vascular resistance, and with shorter 6-min walk distance (p<0.01). Mortality risk doubled for each log higher endostatin (hazard ratio 2.3, 95% CI 1.6–3.4, p<0.001). Endostatin remained an independent predictor of survival when incorporated into existing risk prediction models. Adding endostatin to REVEAL-based and ESC/ERS criteria-based risk assessment strategies improved mortality risk prediction.

Endostatin is a robust, independent predictor of mortality in PAH. Adding endostatin to existing PAH risk prediction strategies improves PAH risk assessment.

## Abstract 2

**Endostatin is a robust, easily accessible biomarker of PAH severity and mortality that is mechanistically related to PAH pathogenesis. Incorporating endostatin into commonly used risk prediction strategies for PAH improves prediction of mortality.** https://bit.ly/3kzGT0w

## Introduction

Pulmonary arterial hypertension (PAH) results from a complex interplay of dysregulated biological pathways, with uncontrolled cellular proliferation, inflammation, autoimmunity and impaired angiogenesis contributing to pulmonary vascular remodelling, right ventricular failure and death [1–3]. The selection and timing of clinical interventions hinges upon serial assessment of disease severity and mortality risk based on a variety of risk prediction strategies. The clinical tools currently available for risk prediction are imperfect, however. Definitive assessment of pulmonary pressures can only be made with right heart catheterisation, which is invasive. Common noninvasive tests suffer a variety of limitations: 6-min walk distance (6MWD) may be confounded by musculoskeletal comorbidities; pulmonary function testing requires consistent patient effort; and echocardiography correlates poorly with invasive haemodynamics, with some echocardiographic measures qualitatively assessed and subject to interpretation.

Serum biomarkers are objective, noninvasive and easily obtained, but to date, only the brain natriuretic peptide and its N-terminal pro-brain natriuretic peptide (NTproBNP) are commonly used in clinical practice for PAH risk stratification. While other serum biomarkers have been robustly associated with the presence of PAH and with clinical outcomes [4, 5], all have intrinsic limitations regarding their specificity for PAH or the right ventricle, as most PAH markers have been studied due to their known relevance in left heart disease or in autoimmune or inflammatory conditions. Right ventricular dysfunction is the major determinant of morbidity and mortality in PAH [3, 6]. Therefore, defining unique noninvasive markers that reflect fundamental PAH or right ventricular pathobiology could allow for more tailored risk assessments.

Endostatin is a circulating angiostatic peptide derived from the protein collagen XVIII, α-1 (COL18A1) that is known to inhibit tumour angiogenesis and coronary collateral formation [7–10]. Previous work by our group has demonstrated that endostatin inhibits pulmonary artery endothelial cell proliferation and migration and promotes endothelial cell apoptosis, fundamental features driving PAH pathobiology [11]. The right ventricular response to increased load imposed by pulmonary vascular change is the major determinant of morbidity and mortality in PAH [3, 6]. Decreased angiogenesis is a feature of right ventricular failure in preclinical models of disease [12], and decreased right ventricular perfusion is observed in clinical PAH [13]. Thus, endostatin may be molecularly relevant to both PAH pathogenesis and right ventricular dysfunction, positioning endostatin as a potential mechanistic biomarker.

Previously published associations between circulating endostatin levels and survival in PAH have been inconsistent, losing significance with adjustment for established disease severity markers, including 6MWD, New York Heart Association Functional Class (NYHA FC) and NTproBNP [14]. However, prior studies were conducted in small single-centre cohorts, limiting multivariable modelling or subgroup analyses. Therefore, the true clinical potential of endostatin as a prognostic biomarker in PAH remains uncertain. In the current study, we examine the prognostic performance of endostatin in PAH in a large, multicentre cohort of PAH subjects. We also sought to assess the value gained by adding endostatin measurements to established PAH risk stratification models. We hypothesised that endostatin would significantly associate with PAH severity, independently predict mortality risk and add value to existing PAH risk prediction models.

## Methods

This observational cohort study was approved by the Johns Hopkins University Institutional Review Board. Serum samples and clinical data from adult subjects with PAH were obtained from the multicentre National Heart, Lung and Blood Institute-sponsored PAH Biobank (www.pahbiobank.org). Subjects provide informed consent and serum samples at the time of enrolment. Methods for data collection and processing serum samples have been previously published [4, 5]. An electrochemiluminescence assay was developed to measure endostatin levels on a 96-well plate from Meso Scale Discovery (Gaithersburg, MD, USA). The full assay protocol has been previously published [5]. The per cent coefficient of variation for endostatin across plates (n=25) was 2.38%.

Cohort demographics and clinical characteristics were summarised with descriptive statistics. To examine associations between endostatin and clinical phenotypes, endostatin served as an independent variable in regressions of continuous clinical variables with adjustments for age and sex. Endostatin levels were right-skewed and natural log-transformed to reduce the influence of outliers. To examine associations of mortality with endostatin dichotomised above *versus* below its median, unadjusted survival analyses were conducted using the Kaplan–Meier method, and adjusted analyses were performed using multivariable Cox proportional hazard models with potential confounders of the relationship between endostatin and survival included as covariates. Covariates were examined for collinearity using variance inflation factors and Pearson correlation coefficients; highly collinear covariates were excluded. Biomarker associations were examined in the overall cohort and in prespecified subgroups of the two predominant PAH subtypes. To assess the value of adding endostatin to existing noninvasive markers and established risk prediction models, extended time-to-event models including endostatin were compared to null models without endostatin using likelihood ratio tests, Akaike and Bayesian information criteria, and C-statistics. Existing risk prediction tools tested as null or base models included the REVEAL 2.0 risk model developed from the Registry to Evaluate Early and Long-term PAH Disease Management and the French Pulmonary Hypertension Registry risk assessment strategy of tabulating the number of low-risk criteria present according to 2015 European Society of Cardiology/European Respiratory Society (ESC/ERS) guidelines [15–17]. Only subjects with sufficient data available to calculate REVEAL 2.0 risk scores and tabulate ESC/ERS low-risk criteria were included in assessments of the respective risk prediction tools. Missing data was considered missing not completely at random, and complete-case analyses were initially performed, followed by additional sensitivity analyses excluding variables with significant missingness. A p-value of <0.05 was considered statistically significant.

## Results

The cohort consisted of 2017 subjects with PAH and was composed primarily of women (80% female) of European ancestry (EA) (82% EA) in the sixth decade of life. Subjects in this prevalent cohort had moderately severe disease, with 45% of the cohort classified as having NYHA FC III or IV symptoms, mean±sd pulmonary arterial pressure (mPAP) of 50±15 mmHg, and pulmonary vascular resistance (PVR) of 10±6 Wood units (table 1). The majority of the cohort was composed of subjects with either idiopathic PAH (IPAH) (n=870) or connective tissue disease-associated PAH (CTD-PAH) (n=623). The median time from PAH diagnosis to cohort enrolment was 48 months (interquartile range (IQR) 14–92 months). For this analysis, time under observation began at cohort enrolment, and the cohort was right-censored in July 2018. A total of 1984 subjects (98.4%) had follow-up data available to ascertain survival. Subjects without follow-up data were excluded from time-to-event analyses. Among these 1984 subjects, 338 deaths occurred over the follow-up period. The median time from enrolment to death or censor was 41 months (IQR 28–55 months).

**TABLE 1:** Demographics and clinical characteristics of the PAH Biobank cohort

**Demographics**	**Overall**	**CTD-PAH**	**IPAH**
**Subjects n**	2017	623	870
**Age years**	55±15	59±14	55±15
**Sex, n female (%)**	1611 (80)	565 (91)	698 (80)
**Genetic ancestry, n EA (%)**	1662 (82)	564 (91)	780 (90)
**Aetiology n FPAH/PVOD/PortoPulm/Congenital/Drug/HIV/Other**	81/8/111/171 /93/42/18		
**NYHA FC, n I/II/III/IV (% III/IV)**	90/451/789/118 (45)	24/140/266/34 (65)	38/188/340/56 (64)
**6MWD m**	347±141	327±160	351±136
**Deaths n (%)**	324 (16)	125 (20)	112 (13)
**Biomarkers**			
Endostatin pg·mL^−1^ median (IQR)	37 515 (27 946–50 901)	41 504 (31 639–55 487)	37 087 (27 856–50 028)
NTproBNP pg·mL^−1^ median (IQR)	672 (217–2164)	907 (331–3077)	520 (183–1621)
**Haemodynamics**			
RAP mmHg	9±5	9±5	9±6
mPAP mmHg	50±15	44±11	51±14
PAWP mmHg	10±4	10±4	10±4
PVR Wood units	10±6	8±5	10±6
Cardiac output L·min^−1^	4.7±1.7	4.7±1.6	4.6±1.6
Cardiac index L·min^−1^·m^−2^	2.7±1.2	2.8±0.9	2.6±1.1
**Therapies n (%)**			
PDE5 inhibitor	1546 (77)	470 (75)	641 (74)
ERA	1205 (60)	370 (59)	515 (59)
IV/SC prostacyclin	699 (35)	161 (26)	355 (41)
CCB	199 (10)	51 (8)	99 (11)

Data are presented as mean±sd, unless otherwise indicated. PAH: pulmonary arterial hypertension; CTD-PAH: connective tissue disease-associated PAH; IPAH: idiopathic PAH; EA: European ancestry; FPAH: familial PAH; PVOD: pulmonary veno-occlusive disease; Portopulm: portopulmonary hypertension; NYHA FC: New York Heart Association Functional Class; 6MWD: 6-min walking distance; IQR: interquartile range; NTproBNP: N-terminal pro-brain natriuretic peptide; RAP: right atrial pressure; mPAP: mean pulmonary arterial pressure; PAWP: pulmonary artery wedge pressure; PVR: pulmonary vascular resistance; PDE5: phosphodiesterase-5; ERA: endothelin receptor antagonist; IV: intravenous; SC: subcutaneous; CCB: calcium channel blocker.

### Endostatin associations with clinical phenotypes and outcomes

Endostatin levels for the overall cohort and IPAH and CTD-PAH subgroups are shown in table 1. Endostatin levels for all PAH subgroups are shown in supplementary table 1. Endostatin was highest in subjects with CTD-PAH and lowest in those with portopulmonary hypertension. Endostatin levels among subjects in both of these subgroups were significantly different from those in the overall cohort.

In the overall cohort, each log higher endostatin was associated with several important disease severity measures, including a 1.84-mmHg higher right atrial pressure (RAP), 1.99 mmHg mPAP and 0.98-Wood unit higher PVR. Additionally, each log higher endostatin was associated with a 5-mL lower stroke volume, 190-mL·mmHg^−1^ lower pulmonary arterial compliance and a 54-m shorter 6MWD (table 2). In subgroup analyses, endostatin associations with RAP, mPAP, PVR and 6MWD were of greater magnitude in IPAH compared to CTD-PAH (supplementary table 2). Cardiac output demonstrated a significant association with endostatin in the IPAH subgroup.

**TABLE 2:** Age- and sex-adjusted endostatin associations with clinical variables in the overall PAH Biobank cohort

**Variable**	**Regression coefficient**
**RAP mmHg**	1.84 (1.35–2.33, <0.001)
**mPAP mmHg**	1.99 (0.75–3.23, 0.002)
**PAWP mmHg**	0.15 (−0.23–0.53, 0.45)
**PVR Wood units**	0.98 (0.44–1.2, <0.001)
**Cardiac output L·min^−1^**	−0.14 (−0.30–0.03, 0.10)
**Cardiac index L·min^−1^·m^−2^**	−0.10 (−0.21–0.01, 0.07)
**Stroke volume L**	−0.005 (−0.008– −0.002, 0.003)
**Pulmonary arterial compliance mL·mmHg^−1^**	−0.19 (−0.31– −0.08, 0.001)
**6MWD m**	−53.5 (−70.7– −36.2, <0.001)
**NTproBNP pg·mL^−1^**	1.12 (1.00–1.25, <0.001)

Values are presented as β-coefficient (95% confidence interval, p-value) per log endostatin. RAP: right atrial pressure; mPAP: mean pulmonary arterial pressure; PAWP: pulmonary artery wedge pressure; PVR: pulmonary vascular resistance; 6MWD: 6-min walking distance; NTproBNP: N-terminal pro-brain natriuretic peptide.

**FIGURE 1:**
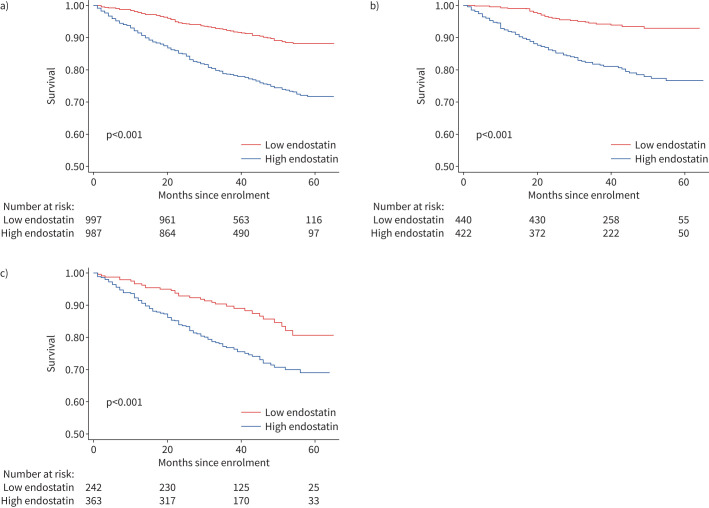
Kaplan–Meier curves depicting associations between endostatin above *versus* below the median and survival in a) the overall cohort, b) idiopathic pulmonary arterial hypertension (IPAH) and c) connective tissue disease-associated PAH (CTD-PAH).

In unadjusted survival analysis, subjects with endostatin above the cohort median experienced worse survival than subjects with lower endostatin levels. The association between high endostatin levels and mortality was also present in unadjusted survival analyses in IPAH and CTD-PAH subgroups (figure 1a–c). In multivariable Cox proportional hazard models adjusted for multiple potential confounders of the relationship between serum endostatin and survival, each log higher endostatin was associated with a roughly two-fold increased risk of mortality (hazard ratio (HR) 2.32, 95% CI 1.56–3.45, p<0.001) (table 3). In the IPAH subgroup, each log higher endostatin was associated with a nearly six-fold increase in mortality (HR 5.68, 95% CI 2.4–12.8, p<0.001). In the CTD-PAH subgroup, the magnitude of the relationship between serum endostatin and mortality was attenuated, and its significance was lost (HR 1.76, 95% CI 0.94–3.32, p=0.08).

**TABLE 3:** Age- and sex-adjusted endostatin associations with survival in the PAH Biobank cohort, overall and by disease subtype

	**Univariable HR**	**Multivariable adjusted HR** **^#^**
**Overall cohort**	3.52 (2.75–4.50, <0.001)	2.32 (1.56–3.45, <0.001)
**CTD-PAH subgroup**	2.91 (1.93–4.38, <0.001)	1.76 (0.94–3.32, 0.080)
**IPAH subgroup**	6.44 (4.16–9.98, <0.001)	5.68 (2.4–12.8, <0.001)

Values are presented as hazard ratio (HR) (95% confidence interval, p-value) per log endostatin. PAH: pulmonary arterial hypertension; CTD-PAH: connective tissue disease-associated PAH; IPAH: idiopathic PAH; NYHA FC: New York Heart Association Functional Class; 6MWD: 6-min walking distance; RAP: right atrial pressure; mPAP: mean pulmonary arterial pressure; PVR: pulmonary vascular resistance. **^#^**: multivariable models are adjusted for age, sex, PAH-specific therapy, NYHA FC, 6MWD, RAP, mPAP, confidence interval and PVR.

### Incorporating endostatin into ESC/ERS criteria-based risk prediction strategies

**FIGURE 2:**
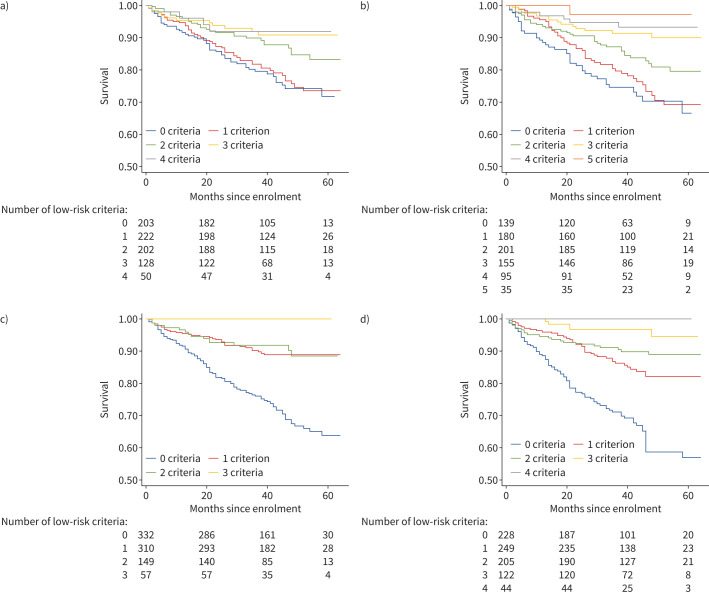
Kaplan–Meier curves depicting survival times by a) number of European Society of Cardiology/European Respiratory Society (ESC/ERS) low-risk criteria applied to the overall cohort, b) number of low-risk criteria in the overall cohort after adding an additional low-risk variable for endostatin less than the cohort median, c) number of noninvasive low-risk criteria in the overall cohort and d) number of noninvasive low-risk criteria in the overall cohort after adding an additional low-risk variable for endostatin less than the cohort median.

820 subjects had complete data available to tabulate ESC/ERS low-risk criteria. Low-risk criteria include functional class (FC) I–II, 6MWD >440 m, RAP <8 mmHg and CI ≥2.5 L·min^−1^·m^−2^ [17, 18]. Adding an additional low-risk variable for endostatin less than the median (37 515 pg·mL^−1^) improved discrimination of risk groups and produced additional mortality risk strata, as shown in figure 2a and b. Mortality differences between subjects with *versus* without low-risk endostatin levels and possessing all four low-risk features are shown in supplementary figure 1a. Differences between subjects with *versus* without low-risk endostatin levels and with no low-risk features are shown in supplementary figure 1b. Univariable and multivariable hazard ratios for each of the low-risk criteria as well as the biomarkers NTproBNP and endostatin are shown in table 4. FC, 6MWD and both biomarkers were significantly predictive of mortality in univariable analysis, though only FC and the two biomarkers NTproBNP and endostatin remained independently predictive after adjustment for all other low-risk parameters.

**TABLE 4:** Univariable and multivariable hazard ratios (HRs) for each of the European Society of Cardiology/European Respiratory Society (ESC/ERS) low-risk parameters and endostatin less than the median

**ESC/ERS low-risk criteria**	**Unadjusted HR (95% CI)**	**p-value**	**Adjusted^#^** **HR (95% CI)**	**p-value**
**Functional Class I–II**	0.51 (0.38–0.68)	<0.001	0.62 (0.41–0.94)	0.024
**6-min walk distance >440 m**	0.40 (0.24–0.66)	<0.001	0.66 (0.36–1.20)	0.174
**RAP <8 mmHg**	0.81 (0.65–1.02)	0.073	0.78 (0.54–1.14)	0.203
**Cardiac index ≥2.5 L·min^−1^·m^−2^**	0.97 (0.78–1.21)	0.778	1.01 (0.71–1.44)	0.955
**NTproBNP <300** **pg·mL^−1^**	0.23 (0.16–0.33)	<0.001	0.27 (0.15–0.47)	<0.001
**Endostatin <median**	0.36 (0.28–0.46)	<0.001	0.42 (0.28–0.64)	<0.001

RAP: right atrial pressure; NTproBNP: N-terminal pro-brain natriuretic peptide. **^#^**: adjusted for all other variables including biomarkers (NTproBNP <300 pg·mL^−1^ and endostatin <median).

A multivariable time-to-event model comprising the four ESC/ERS low-risk categorical variables was significantly improved by adding a variable for endostatin dichotomised at the median (likelihood ratio Chi-squared test 33.78, p<0.001) (supplementary table 3). The French Pulmonary Hypertension Registry also assessed a simplified risk scheme composed of three noninvasive variables (FC I–II, 6MWD >440 m and NTproBNP <300 ng·mL^−1^) and found that this simplified scheme also clearly discriminated mortality risk groups among subjects at follow-up [17]. In our cohort, adding a variable for endostatin dichotomised at the median to the three noninvasive ESC/ERS variables again improved discrimination of risk groups (figure 2c and d) and model fit (likelihood ratio Chi-squared test 19.73, p<0.001) (supplementary table 3).

To assess the relative utility of models based on invasive *versus* noninvasive ESC/ERS risk parameters *versus* models based on biomarkers alone in our cohort, a variety of ESC/ERS criteria-based time-to-event models were constructed and compared using Akaike Information Criteria (AICs) and Harrell's C-statistics in an exploratory analysis. AICs are a means of assessing the relative quality of predictive models by balancing goodness of model fit with model parsimony. The preferred model minimises AIC, that is, it provides the best possible model fit (*e.g.* the least information loss about the underlying data) with the fewest possible model terms. Harrell's C-statistic assesses model ability to discriminate censored outcomes such as survival times, with a C-statistic of 0.5 representing a completely uninformative model, and a C-statistic of 1.0 representing a perfectly informative model. In the overall cohort, a biomarker-only model composed of NTproBNP and endostatin dichotomised at their respective medians outperformed other models by both means of assessment (lowest AIC 1683, C-statistic 0.73), including the full low-risk criteria model, the noninvasive low-risk criteria model, and a low-risk criteria model with both NTproBNP and endostatin added (supplementary table 4).

### Incorporating endostatin into REVEAL-based risk prediction strategies

**FIGURE 3:**
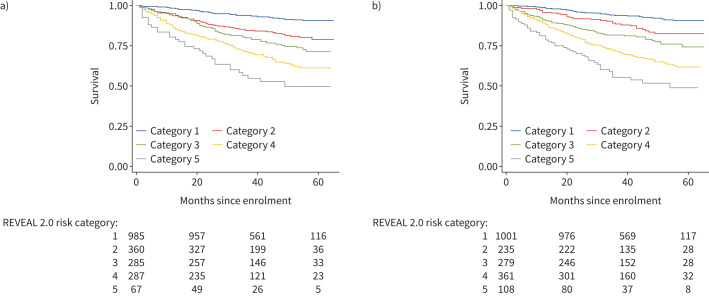
Kaplan–Meier curves depicting survival times by REVEAL 2.0 risk categories based on a) unmodified tabulation of REVEAL risk scores (RRS) and b) tabulation of RRS modified by adding −1 for endostatin below median and +1 for endostatin above median.

One thousand nine hundred and 84 subjects had sufficient data to tabulate REVEAL 2.0 risk scores. When REVEAL 1.0 variable cut points were revised for REVEAL 2.0, two new variables (hospitalisation within 6 months and estimated glomerular filtration rate) were added to the calculator [15]. These new variables are not available in our dataset; however, REVEAL was designed for practical use with the clinical data available at any given point in time and has been shown to maintain its predictive power and calibration when at least seven evaluable parameters are available [15, 19]. Therefore, REVEAL risk scores (RRS) were tabulated for each subject as long as at least seven evaluable parameters were available for a given subject. Methods for tabulating RRS and dividing risk scores into previously defined risk categories have been previously published [5, 20, 21]. The mean±sd RRS for this cohort was 7.31±2.39, and survival curves for each REVEAL risk category are shown in figure 3a. Modification of the RRS by subtracting a point for endostatin below the median and adding a point for endostatin above the median improved separation among lower risk categories in the first 12–24 months of person-time under observation (figure 3b).

Univariable and multivariable hazard ratios for each of the categorical REVEAL 2.0 risk parameters as well as endostatin are shown in table 5. The majority of REVEAL variables were predictive of mortality in univariable analysis; however, in multivariable analysis, only NTproBNP >1100 pg·mL^−1^ and endostatin above the median remained independently predictive after adjustment for all other REVEAL parameters. Among subjects with complete data available for every one of the categorical REVEAL 2.0 parameters available in the cohort (n=438), adding a variable for endostatin dichotomised at the median improved model fit (likelihood ratio Chi-squared test 10.51, p<0.001) (supplementary table 5). Sensitivity analyses were performed to systematically exclude variables with significant missingness, and for each model comparison, addition of endostatin significantly improved model fit (supplementary table 5).

**TABLE 5:** Univariable and multivariable hazard ratios (HRs) for each of the categorical REVEAL 2.0 risk parameters and endostatin greater than the median

**REVEAL parameters**	**Unadjusted HR (95% CI)**	**p-value**	**Adjusted^#^** **HR (95% CI)**	**p-value**
**CTD-PAH**	1.51 (1.21–1.89)	<0.001	0.90 (0.57–1.44)	0.673
**Portopulmonary hypertension**	2.19 (1.54–3.11)	<0.001	1.84 (0.81–4.21)	0.146
**Heritable PAH**	0.65 (0.34–1.27)	0.207	1.44 (0.33–6.21)	0.627
**Male >60 years**	1.94 (1.40–2.69)	<0.001	0.79 (0.37–1.71)	0.554
**NYHA/WHO**				
FC I	0.59 (0.25–1.37)	0.222	0.27 (0.04–2.08)	0.210
FC II	ref		ref	
FC III	1.82 (1.34–2.47)	<0.001	1.00 (0.55–1.82)	0.997
FC IV	1.92 (1.20–3.09)	0.007	0.97 (0.41–2.30)	0.951
**SBP <110 mmHg**	0.71 (0.48–1.03)	0.069	0.81 (0.42–1.55)	0.518
**Heart rate >96 beats per min**	0.77 (0.50–1.20)	0.255	0.63 (0.29–1.34)	0.231
**6MWD**				
<165 m	1.75 (1.12–2.71)	0.013	1.28 (0.71–2.32)	0.415
165−<320 m	ref		ref	
320−<440 m	0.56 (0.39–0.80)	0.002	0.69 (0.41–1.18)	0.178
≥440 m	0.35 (0.21–0.59)	<0.001	0.45 (0.18–1.11)	0.082
**NTproBNP**				
<300 pg·mL^−1^	0.49 (0.32–0.74)	0.001	0.52 (0.21–1.25)	0.145
300−<1100 pg·mL^−1^	ref		ref	
≥1100 pg·mL^−1^	3.10 (2.35–4.08)	<0.001	3.16 (1.70–5.89)	<0.001
**RAP >20 mmHg**	1.93 (1.23–3.04)	0.004	0.93 (0.40–2.12)	0.861
**PVR <5 Wood units**	1.08 (0.82–1.43)	0.57	1.15 (0.64–2.04)	0.641
**Endostatin**				
<median	ref		ref	
>median	2.76 (2.17–3.51)	<0.001	2.47 (1.39–4.39)	0.002

PAH: pulmonary arterial hypertension; CTD-PAH: connective tissue disease-associated PAH; IPAH: idiopathic PAH; NYHA FC: New York Heart Association Functional Class; WHO: World Health Organization; SBP: systolic blood pressure; 6MWD: 6-min walking distance; NTproBNP: N-terminal pro-brain natriuretic peptide; RAP: right atrial pressure; PVR: pulmonary vascular resistance. **^#^**: adjusted for all other REVEAL variables plus endostatin.

For symmetry with the exploratory analysis conducted to assess the relative utility of various ESC/ERS-based models, a variety of REVEAL-based time-to-event models were constructed and compared using AICs and Harrell's C-statistics (supplementary table 6). Models based on categorical REVEAL variables, tabulated RRS, REVEAL risk categories and biomarkers alone were tested among subjects with complete data available for REVEAL 2.0 parameters. A model including NTproBNP (parameterised per REVEAL 2.0 cutpoints) and endostatin greater than the median was the best fit, most parsimonious model. A model that added a variable for endostatin greater than the median to REVEAL 2.0 categorical parameters was the most discriminatory, with C-statistic 0.79.

## Discussion

To our knowledge, this is the largest study to examine the angiostatic protein endostatin as a biomarker of disease severity and survival in PAH. Our findings demonstrate significant associations between circulating endostatin levels and important measures of disease severity, including haemodynamics and 6MWD. More importantly, our results show strong, significant associations between endostatin levels and mortality, particularly in IPAH, even with adjustment for potential confounders and other disease severity markers. Collectively, these results show that endostatin has clear potential for clinical use as a robust prognostic biomarker in PAH.

Adding information about endostatin measurements to established PAH risk prediction models improved mortality risk stratification and discrimination. Both ESC/ERS criteria-based and REVEAL 2.0 parameter-based models performed well in our cohort; however, we showed that adding endostatin variables to both risk prediction strategies improved predictive capacity. Furthermore, our analyses demonstrated that unlike many other parameters, biomarker measurements remained strong, independent predictors of survival after adjustment for all other variables included in either risk prediction strategy. Noninvasive and biomarker-only models outperformed (in the case of ESC/ERS-based models) or were similarly informative to (in the case of REVEAL-based models) risk prediction models requiring many more variables, many of which require challenging or invasive means to obtain. Taken together, these results suggest that novel markers like endostatin could form the cornerstone of future refinements to PAH risk assessment strategies based on noninvasive parameters only.

Our results build upon previous work by our group that indicated endostatin triggers an angiostatic signal cascade propagated by thrombospondin-1 (TSP-1), inhibitor of differentiation/DNA binding-1 (ID1), and bone morphogenetic protein receptor-2 (BMPR2), which have been directly implicated in the pathogenesis of PAH, to inhibit pulmonary endothelial cell migration, proliferation and cell survival [11, 22]. In addition to effects on the pulmonary vasculature, there is mounting evidence to support a critical role for angiostasis in right ventricular dysfunction and the transition to decompensated right ventricular failure. In a monocrotaline rat model of PAH, the transition to right ventricular failure is marked by a decrease in angiogenic factors and diminished angiogenesis [12]. In humans, decompensated right ventricular failure is associated with greater impairment in angiogenesis than is adaptive right ventricular remodelling [23]. Cardiac MRI has shown that clinical right ventricular dysfunction is associated with reduced right ventricular myocardial perfusion reserve in PAH, further suggesting that a failure of angiogenesis to maintain proper myocyte-microvascular balance contributes to right ventricular failure [13]. Furthermore, reduced myocardial perfusion reserve is associated with increased mortality in PAH [24]. Elevated endostatin has been observed in the remodelled pulmonary vessels in PAH [25], and studies in coronary artery disease have shown levels of endostatin in the serum and pericardium correlate with impaired coronary collateral formation [9, 10, 26]. Importantly, no studies have directly implicated endostatin as a regulator of pulmonary vascular or myocardial angiogenesis, and further basic studies are needed to parse the potential effects of endostatin in pulmonary vasculature *versus* right ventricular myocardium. However, should endostatin prove a direct contributor to either pulmonary vascular or right ventricular pathophysiology, this could lend endostatin a degree of disease specificity not captured by NTproBNP, which reflects nonspecific myocyte stretch.

This study has some limitations. The PAH Biobank does not capture imaging data, and therefore right ventricular metrics are not available to directly examine endostatin associations with right ventricular function. Our dataset did not capture two newer variables incorporated into REVEAL 2.0, and some REVEAL parameters have missing data; however, REVEAL was intended to accommodate missing data, and our results were consistent across several sensitivity analyses excluding variables with significant missingness. While REVEAL and ESC/ERS risk strategies have been tested across multiple cohorts, risk prediction models incorporating endostatin will require external validation. Our analyses do not clarify why endostatin exhibits superior performance characteristics in IPAH *versus* CTD-PAH. There may be unknown contributors to shortened survival times in CTD-PAH that are not represented in our dataset or accounted for in our models, resulting in residual confounding, or this may simply reflect intrinsic differences in biology between the two predominant PAH subgroups. Finally, we do not have data on subject comorbidities, which may impact survival times, in order to adjust for this important variable in our models.

This study has several strengths. This is the largest study to examine an angiostatic factor as a biomarker in PAH, and our results emphasise the importance of including markers representative of biologically relevant pathways in PAH risk assessment. This is a large, well-phenotyped cohort drawing from multiple pulmonary hypertension referral centres. The large size of the PAH Biobank and the detailed clinical data captured allows for multivariable modelling to adjust for key confounders. Similarly, the large sample sizes available for the two predominant PAH subtypes allow for meaningful subgroup analyses.

In conclusion, endostatin is a robust prognostic biomarker that is independently predictive of mortality in PAH and may reflect important aspects of disease pathobiology. Endostatin enhances mortality risk prediction when incorporated into existing risk prediction strategies. This study demonstrates clear potential for endostatin to be incorporated into updated risk stratification tools for PAH. Future studies are needed to determine the potential for endostatin to serve as a diagnostic or serial biomarker, and to clarify genetic, molecular and cellular mechanisms. Should future mechanistic studies implicate endostatin in the causal pathway for PAH development or progression, it may be a plausible target for drug development efforts.

## Supplementary material

10.1183/23120541.00378-2021.Supp1 **Please note:** supplementary material is not edited by the Editorial Office, and is uploaded as it has been supplied by the author.
Supplementary material 00378-2021.SUPPLEMENT
