# Supplementary material for: The angiostatic peptide endostatin enhances mortality risk prediction in pulmonary arterial hypertension
Source: ERJ Open Res. 2021 Oct 11;7(4):00378-2021. doi: 10.1183/23120541.00378-2021 (PMC8503279; doi:10.1183/23120541.00378-2021)
Supplement: Supplementary file 1 [file 00378-2021.SUPPLEMENT.pdf]

Online Data Supplement

The angiostatic peptide endostatin enhances mortality risk prediction in PAH

Catherine E. Simpson, MD MHS, Megan Griffiths, MD, Jun Yang, PhD, Melanie K. Nies, MD, R. Dhananjay Vaidya, PhD, Stephanie Brandal, MS, Lisa J. Martin, PhD, Michael W. Pauciulo, MBA, Eric D. Austin, MD MSCI, D. Dunbar Ivy, MD, William C. Nichols, PhD, Allen D. Everett, MD, Paul M. Hassoun, MD, and Rachel L. Damico, MD PhD

Supplemental Figure Legends

Supplemental Figure 1a. Kaplan-Meier plot representing mortality differences in subjects with versus without low-risk endostatin levels AND all four ESC/ERS low-risk features.

Supplemental Figure 1b. Kaplan-Meier plot representing mortality differences in subjects with versus without low-risk endostatin levels with none of the four ESC/ERS low-risk features.

Supplemental Table 1. Endostatin levels by PAH subgroup

PAH subgroup	Endostatin level	p-value
CTD-PAH	41,504 (31,639-55,487)	<0.01
IPAH	37,087 (27,856-50,028)	0.40
FPAH	32,574 (27,395-44,020)	0.03
PVOD	32,566 (24428-42336)	0.30
Portopulmonary Hypertension	30,357 (19,332-47,385)	<0.01
CHD-APAH	32,951 (23,717-43,830)	0.40
Drug/Toxin-Associated PAH	39,259 (28,520-51,322)	0.63
HIV-APAH	32,894 (23,110-44,520)	0.04
Other	36,014 (22,279-45,419)	0.39

Definition of abbreviations: CTD-PAH: connective tissue-associated PAH; IPAH: idiopathic PAH; FPAH: familial PAH; PVOD: pulmonary veno-occlusive disease; CHD-APAH: congenital heart disease-associated PAH; HIV-APAH: HIV-associated PAH.
Values are presented as median (interquartile range).
p-values reflect rank-sum differences between subjects in each indicated subgroup versus all others.

Supplemental Table 2. Age- and Sex-adjusted ES Associations with Clinical Variables in the PAH Biobank Cohort, by disease subtype

Variable	CTD-PAH	IPAH
**RAP, mmHg**	1.51 (0.65-2.34, 0.001)	2.58 (1.74-3.41, <0.001)
**mPAP, mmHg**	2.97 (1.13-4.80, 0.002)	3.73 (1.70-5.75, <0.001)
**PAWP, mmHg**	-0.649 (-1.33-0.03, 0.06)	0.64 (0.02-1.26, 0.04)
**PVR, Wood units**	1.45 (0.65-2.26, <0.001)	1.54 (0.65-2.42, 0.001)
**Cardiac output, L/min**	-0.03 (-0.29-0.24, 0.85)	-0.30 (-0.55- -0.04, 0.023)
**Cardiac index, L/min/m^2**	-0.08 (-0.23-0.06, 0.27)	-0.15 (-0.33- 0.02, 0.09)
**Stroke Volume, L**	-0.001 (-0.006- 0.003, 0.57)	-0.006 (-0.011- -0.002, 0.009)
**Pulmonary arterial compliance, ml/mmHg**	-0.24 (-0.47- -0.01, 0.042)	-0.23 (-0.41- -0.06, 0.008)
**6MWD, m**	-38.8 (-74.8- -28.0, 0.03)	-68.5 (-95.1- -41.9, <0.001)

Values are presented as beta coefficient (95% confidence interval, p value) per log ES. CTD-PAH: connective tissue disease-associated PAH; IPAH: idiopathic PAH. See Table 1 for all other abbreviations.

Supplemental Table 3. Statistical Testing of Extended ESC/ERS Survival Models with Endostatin Added Compared to Null Models without Endostatin with Likelihood Ratio Tests

ESC/ERS Null Models	Extended Models	LR X^2	*p* value
4 low risk criteria*	4 low risk criteria + ES***	33.78	<0.001
Noninvasive low risk criteria**	Noninvasive low risk criteria + ES	19.73	<0.001

Definition of abbreviations. LR $X^{2:}$ likelihood ratio chi-squared.
*Low risk criteria are FC I-II, 6MWD >440m, RAP<8mmHg, and CI ≥ 2.5 L/min/m2
**Noninvasive criteria are FC I-II, 6MWD >440m, NTproBNP <300pg/mL
***ES < median 37515 pg/mL

Supplemental Table 4. Statistical Testing of ESC/ERS-Based Survival Models with AICs, BICs, and C-statistics

ESC/ERS-based model variables	Degrees of freedom	AIC	BIC	C-statistic
4 low risk criteria*	4	1763.436	1782.200	0.6166
4 low risk criteria + ES	5	1731.659	1755.113	0.6848
4 low risk criteria + NTproBNP	5	1722.46	1745.914	0.6904
4 low risk criteria + ES + NTproBNP	6	1705.497	1733.642	0.7183
Noninvasive low risk criteria**	3	1721.489	1735.562	0.6791
Noninvasive low risk criteria + ES	3	1703.224	1721.988	0.7118
NTproBNP <300pg/mL	1	1736.895	1741.586	0.6253
ES < median***	1	1737.830	1742.521	0.6445
NTproBNP <300pg/mL + ES < median	2	1710.770	1720.151	0.6949
NTproBNP < median****	1	1699.755	1704.446	0.6876
NTproBNP <median + ES < median	2	**1683.201**	**1692.582**	**0.7283**

Definition of abbreviations. AIC: Akaike information criteria; BIC: Bayesian information criteria.
*Low risk criteria are FC I-II, 6MWD >440m, RAP<8mmHg, and CI ≥ 2.5 L/min/m2
**Noninvasive low risk criteria are FC I-II, 6MWD >440m, NTproBNP <300 pg/mL
***ES median 37514.91 pg/mL
**** NTproBNP median 672.559 pg/mL
Lowest AIC/BIC and highest C-statistic are bolded

Supplemental Table 5. Statistical Testing of Extended REVEAL 2.0 Models with Endostatin Added Compared to Null Models without Endostatin with Likelihood Ratio Tests

REVEAL Null Models	Extended Models	LR X^2	*p* value	N
REVEAL parameters	REVEAL parameters + ES>median	10.51	0.001	438
**Sensitivity analyses:**				
REVEAL parameters, except 6MWD	REVEAL parameters, except 6MWD + ES>median	16.98	<0.001	670
REVEAL parameters, except HR and SBP	REVEAL parameters, except HR and SBP + ES>median	7.51	0.023	787
REVEAL parameters, except NYHA FC	REVEAL parameters, except NYHA FC + ES>median	11.34	0.003	520

Definition of abbreviations. LR $X^{2:}$ likelihood ratio chi-squared. See Table 1 for other abbreviations.
REVEAL parameters: presence of CTD-PAH, portopulmonary hypertension, heritable PAH; male > 60 years NYHA/WHO FC I, FC II,FC III,FC IV; SBP <110 mmHg; HR >96 beats per minute; 6MWD <165m, 165- <320m, 320- <440m, ≥440m; NTproBNP <300pg/mL, 300- <1100pg/mL, ≥1100pg/mL; RAP > 20mmHg; PVR < 5 Wood units

Supplemental Table 6. Statistical Testing of REVEAL-Based Survival Models with AICs, BICs and C-statistics for subjects without any missing data for REVEAL parameters (n=438)

REVEAL-based model variables	Degrees of freedom	AIC	BIC	C-statistic
REVEAL 2.0 parameters	16	966.413	1031.728	0.7828
REVEAL 2.0 parameters + ES>median	17	957.8997	1027.297	**0.7932**
REVEAL 2.0 risk scores	11	994.5804	1039.485	0.6999
REVEAL 2.0 risk scores + ES>median	13	982.1773	1035.246	0.7374
REVEAL 2.0 risk categories	4	987.8481	1004.177	0.6831
REVEAL 2.0 risk categories + ES	4	972.0048	988.3337	0.7205
REVEAL 2.0 NTproBNP	2	960.4595	968.6239	0.7256
ES > median	1	988.2197	992.302	0.6653
REVEAL 2.0 NTproBNP + ES > median	3	**946.4291**	**958.6758**	0.7590
NTproBNP > median	1	970.6574	974.7396	0.6949
NTproBNP >median + ES >median	2	953.8023	961.9668	0.7428

REVEAL parameters: presence of CTD-PAH, portopulmonary hypertension, heritable PAH; male > 60 years NYHA/WHO FC I, FC II,FC III,FC IV; SBP <110 mmHg; HR >96 beats per minute; 6MWD <165m, 165- <320m, 320- <440m, ≥440m; NTproBNP <300pg/mL, 300- <1100pg/mL, ≥1100pg/mL; RAP > 20mmHg; PVR < 5 Wood units; REVEAL risk scores range 1-15; REVEAL risk categories range 1-5.

Lowest AIC/BIC and highest C-statistic are bolded

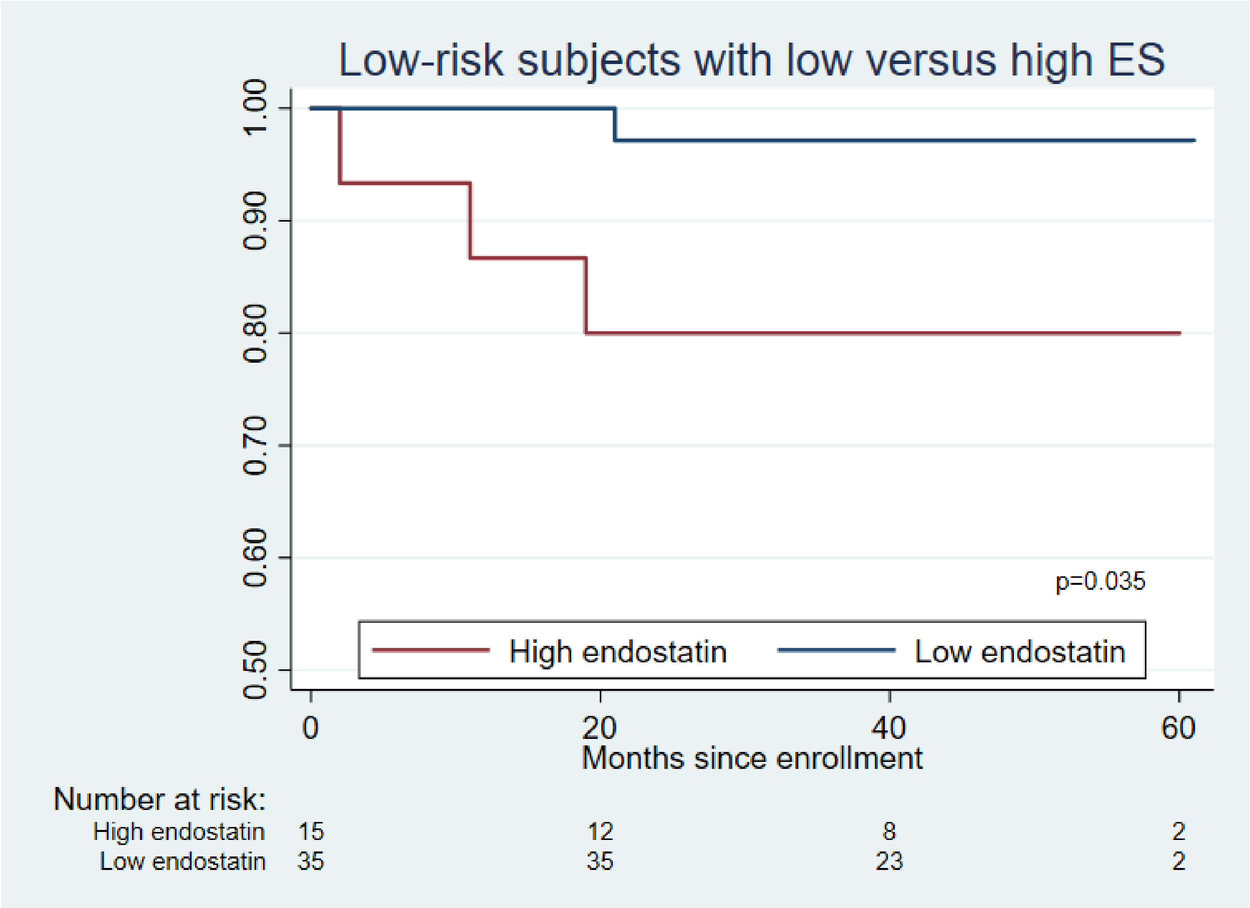

Supplemental Figure 1a

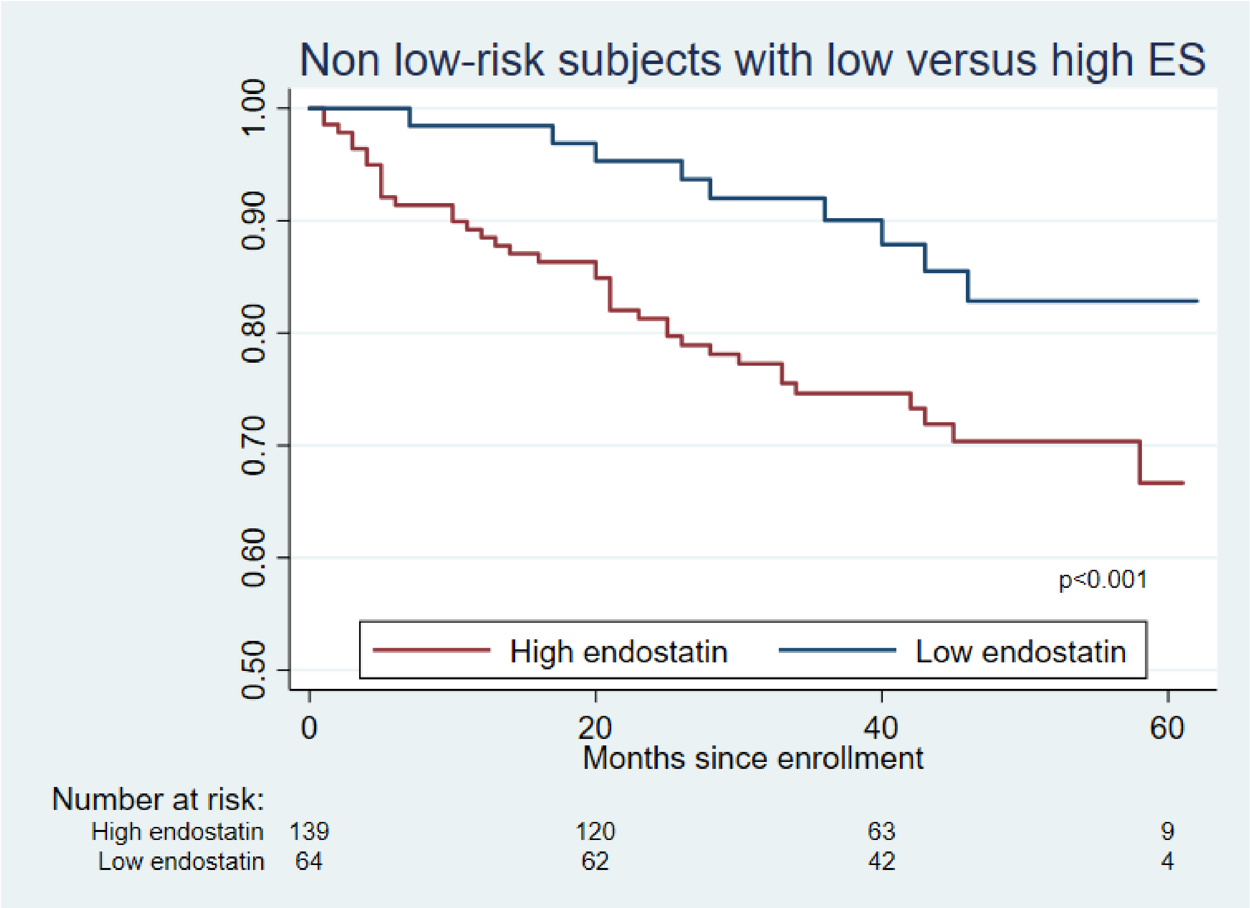

Supplemental Figure 1b.
